# Insights into the Effects of Insecticides on Aphids (Hemiptera: Aphididae): Resistance Mechanisms and Molecular Basis

**DOI:** 10.3390/ijms24076750

**Published:** 2023-04-04

**Authors:** Rana Muhammad Kaleem Ullah, Fukun Gao, Aatika Sikandar, Haiyan Wu

**Affiliations:** Guangxi Key Laboratory of Agric-Environment and Agric-Products Safety, College of Agriculture, Guangxi University, Nanning 530004, China; ranakaleem193@gmail.com (R.M.K.U.); 18854881887@163.com (F.G.); aatika_sikander@yahoo.com (A.S.)

**Keywords:** aphids, enzymatic machinery, gene mutation, resistance mechanisms, RNA-Seq technique, insecticide resistance

## Abstract

With the passage of time and indiscreet usage of insecticides on crops, aphids are becoming resistant to their effect. The different classes of insecticides, including organophosphates, carbamates, pyrethroids and neonicotinoids, have varied effects on insects. Furthermore, the molecular effects of these insecticides in aphids, including effects on the enzymatic machinery and gene mutation, are resulting in aphid resistance to the insecticides. In this review, we will discuss how aphids are affected by the overuse of pesticides, how resistance appears, and which mechanisms participate in the resistance mechanisms in various aphid species as significant crop pests. Gene expression studies were analyzed using the RNA-Seq technique. The stress-responsive genes were analyzed, and their expression in response to insecticide administration was determined. Putative insecticide resistance-related genes, cytochrome P450, glutathione S-transferase, carboxylesterase CarEs, ABC transporters, cuticle protein genes, and trypsin-related genes were studied. The review concluded that if insecticide-susceptible aphids interact with ample dosages of insecticides with sublethal effects, this will result in the upregulation of genes whose primary role is to detoxify insecticides. In the past decade, certain advancements have been observed regarding insecticide resistance on a molecular basis. Even so, not much is known about how aphids detoxify the insecticides at molecular level. Thus, to attain equilibrium, it is important to observe the manipulation of pest and insect species with the aim of restoring susceptibility to insecticides. For this purpose, this review has included critical insights into insecticide resistance in aphids.

## 1. Introduction

Aphids (Hemiptera: Aphididae) are significant economic pests that are found all over the globe. They impose substantial economic losses by directly feeding on plants, spreading plant viruses, and producing honeydew [1,2]. They are major insect pests of various plants, including alfalfa [3], wheat [4], potato [2,5,6] sugar beet and tobacco. More than 50 families of plant are affected by this highly polyphagous pest [7,8,9]. On infection, they consume sap from phloem tissue, which depletes plant nutrients, stunts development, lowers yield, and destroys plants [3]. However, their management is challenging because the mobility of aphids is extremely high and they reproduce rapidly and in large numbers, which enables them to swiftly invade and harm plants [10]. Almost all aphid species can be controlled with chemical insecticides. It is one of the most prevalent management strategies employed so far. The most commonly used insecticides are organophosphates, carbamates, pyrethroids, cyclodienes, and neonicotinoids [11].

However, excessive aphicide usage, particularly in populations of the species *Aphis gossypii*, *Myzus persicae*, *Metopolophium dirhodum*, and *Aphis fabae*, may result in high levels of pesticide resistance [12,13,14,15]. *Rhopalosiphum padi* and *Sitobion avenae*, two species of wheat aphid, have developed different degrees of resistance to pesticides with various mechanisms of action, according to earlier research [16,17], which makes control considerably more difficult [10]. Resistance allows for higher insecticide application rates and higher doses, lower yields, and, eventually, higher risks of residues in food. It also causes environmental harm owing to adverse effects on beneficial organisms as well as an increase in the emission of xenobiotics. In addition, the primary targets of resistance are important body mechanisms including nerve impulse transmission, lipid biosynthesis, and cellular respiration [18,19]. According to the available statistical figures, there are at least 20 aphid species—including the invasive soybean aphid (*Aphis glycines*)—and as many as 40 aphid species that are known to display resistance against several insecticide groups [20,21]. Considering the critical need for farming practices aimed at maximizing the yield of crops, pesticides from the following groups been used successfully to chemically manage aphids worldwide: organophosphates, pyrethroids, neonicotinoids, and carbamates. For the reasons outlined above, it is crucial to search for insensitive populations and identify which resistance mechanisms are preferred in order to discover novel strategies for restricting the development of their spread.

The cotton aphid, *A. gossypii*, is a prominent pest for a variety of agriculturally significant crops. This aphid causes damage by transmitting viruses while feeding. In China, pesticide use is a common method for controlling cotton aphids; these treatments are often repeated annually. On the other hand, due to the extensive use of insecticides such as carbamates, pyrethroids, and organophosphates, cotton aphids have developed a resistance to these pesticides. From 1992 to 1998, cotton aphids in the Xinjiang (Uygur Autonomous Region) have shown varying degrees of pesticide resistance [22]. Resistant aphids have been seen in the main cotton growing areas of Xinjiang, Shandong, Hebei, Sichuan, Henan, Shanxi, Liaoning, Shaanxi, and Beijing [23].

One of the most important pests that has the capability of targeting a wide variety of 40 plant families of horticultural crops worldwide is named “green peach aphid”, *Myzus persicae* (Sulzer) [2,8]. It is known that different vegetables, such as tomatoes, eggplants, and capsicums, can face considerable damage from *M. persicae*. Additionally, cruciferous vegetables, and several pulse and canola crops are particularly affected by *M. persicae* in Australia. The feeding mechanism of aphids includes sucking sap from buds and leaves; however, when populations are enormous, aphids, causing diminished or stunted development in new plants, can blanket the whole crop canopy. *M. persicae*, similar to all other aphids, secretes honeydew, which causes subsequent fungal infection, limiting photosynthesis and compromising plant development and crop market value [2,24]. *M. persicae* has also been linked to the transmission of 100 plant viruses, including beet cucumber mosaic virus, western yellow virus, and potato leafroll virus [25,26].

From 2017–2019, the susceptibility of 19 different strains of *M. dirhodum* from 7 provinces in Nothern China was calculated for macrolide (abamectin), organophosphates, pyrethroids, and neonicotinoids. Two populations showed resistance to thiamethoxam, with RLRs (relative resistance ratios) of 134.03 and 103.03; however, one population was beta-cypermethrin-resistant, with an RLR of 121.42. Using RLR as a baseline, resistance to imidacloprid was indicated by an RLR of 1.50 to 57.29, resistance to abamectin was indicated by an RLR of 1.10 to 25.89, and resistance to omethoate was indicated by an RLR of 1.07 to 18.73, varying within this range from being susceptible to displaying moderate resistance. Nonetheless, they varied in their susceptibility to bifenthrin with an RLR of 1.14 to 6.02, demonstrating tolerance or minimal degrees of resistance. The LC50 for thiamethoxam and beta-cypermethrin using pair-wise correlation analysis also illustrates how these two pesticides are resistant to one another [14].

Chemicals are increasingly being used to manage *M. persicae* in a variety of food crops across the world. This aphid species is now widely targeted with pesticides in Australia’s vegetable crops, oilseed, and pulses [27], and is a major cause of development of insecticide-resistance in the populations of *M. persicae* [5,28]. Worldwide, 74 pesticides from different chemical classes, including neonicotinoids, carbamates, organophosphates, organochlorines, and pyrethroids, have been documented to be resistant [29]. By taking advantage of recent advances in this field, we were able to gain a clearer understanding of the degree to which aphids are resistant to various insecticides currently. Scientists will be able to compare how various aphid species from all over the world have evolved their resistance to various insecticides. This will help us in identifying a possible selection target for better aphid pest management and to discover the mechanisms involved in the development of insecticide resistance in aphids with the goal of restoring pesticide effectiveness.

## 2. Insecticide Resistance in Aphids to Different Insecticides

### 2.1. Organophosphate Resistance

Organophosphates (Ops) were first used as insecticides over 80 years ago for pest control management, according to documents. Today, organophosphates constitute 30% of all synthetic pesticides approved for use in the United States. Their usage may vary with settings [30]. The mechanism of action of organophosphates includes toxicity caused by the inhibition of acetylcholinesterase (AChE). AChE causes deterioration of the excitatory neurotransmitter acetylcholine, leading to a termination of nerve impulse transmission at the synapses. Inhibition of the functioning of the enzyme acetylcholine, if prolonged for a sufficient duration at synapses, results in hyperexcitation and then, eventually, death of insect pest. In the codling moth, previous works suggested that carboxylesterases (CarEs) are associated with detoxification of xenobiotics, especially insecticides. Previous research on the codling moth *Cydia pomonella* (L.) showed that carboxylesterases were linked to detoxifying of xenobiotics, particularly insecticides [31,32,33,34,35,36], and most researchers concur that variations in CarEs activity are linked to resistance in many insect species to organophosphates (Ops), synthetic pyrethroids (SPs), and carbamates (CBs) [36,37]. The manner in which CarEs are expressed in insects may indicate its possible role in the codling moth [38]. Different species of aphids, such as *Aphis gossypii*, *Myzus persicae*, *Aphis fabae* and *Rhopalosiphum padi*, have been reported to develop resistance against organophosphates (methmidophos, monocrotophos, and phosphamidon). Resistance to organophasphates in aphids ranges from low (1-fold) to moderately high levels (85-fold) [39,40,41], as shown in Table 1.

#### Mechanism of Organiphosphate Resistance

Pesticide intoxication contains multiple types of pharmacological interfaces: obstacles to penetration, enhanced metabolic detoxification, and abnormal molecular association with the insecticide at its final target site. Thus, insect and pest tolerance to insecticides relies on the combination of a lethal molecule with a target site and the pharmacokinetic mechanisms that determine the pace and amount of toxin supplied to the target site. Considering these compounds’ diverse chemical compositions, there are very few recognized modes of pesticide resistance [51]. These processes have been categorized in a variety of different ways, but they all involve either penetration barriers, increased metabolic detoxification, or sensitivity to the target position or site. The modes of tolerance to organophosphate pesticides are comparable to those of insecticide resistance, which has been discussed by a number of scholars [51,52,53,54,55]. Some of the characteristic properties of organophosphates, the mechanism of organophosphate resistance, and the interactions between resistance mechanisms in diverse pest species are considered in this review.

Organophosphate pesticides have unique structural characteristics that allow biotransformation sites for activation as well as detoxification. Major resistance in aphids comes from metabolic enzymes of phase-I and phase-II, viz. carboxylesterases and AChE genes (*Ace1* and *Ace2*). Several mutations have been found in association with organophosphate resistance, i.e., A128V, H104R, and T333P, and Thr^210^→Met^210^, Asn^294^→Lys^294^, Gly^408^→Asp^408^, and Ser^441^→Phe^441^ mutations in carboxylesterases. The enzyme structures of A302S, F139L, F368(290)L S329(228)P, and V435(356)A in *Ace1* and *Ace2* are shown in Table 2 [41,56,57,58].

In addition to these, Ops resistance is often caused by cytochrome P450 monooxygenases [71,72,73]. Heme-containing proteins known as cytochrome P450s are found across all cellular entities and are microsomal (endoplasmic reticulum-bound) within eukaryotes. P450s catalyze oxidative processes, including hormone metabolism and harmful external substances such as pesticides and secondary plant chemicals, using the cofactor NADPH. These oxidations produced highly polar molecules that have a biological purpose (like hormones) and are more easily reversed by parallel conjugation enzymes. P450 inducers may be substrates or not, and some P450 inducers act as catalyzers, whereas others do not [73,74].

### 2.2. Pyrethroid Resistance

The literature from several Asian countries helps in understanding the “light” extent of field-evolved soybean aphid resistance to the pesticide class of organophosphates [75,76]. In addition, in the United States, researchers [75] observed no resistance to pyrethroids, organophosphates, or to any other insecticide class, such as neonicotinoids, in Michigan’s 2007–2008 field-collected soybean aphid population. The resistance ratios of North Central Zone soybean aphid populations to neonicotinoids, notably thiamethoxam, were greater than 20-fold. This can be categorized as “moderate resistance” [77].

Currently, we do not have information regarding neonicotinoids’ failure in controlling the soybean aphid from the samples collected from the field. The first clue that soybean aphids are resistant to insecticides are claims that in North America, these pesticides have completely failed to suppress the pest population [21]. From 2015 to 2016, soybean aphids showed 40-fold resistance ratios against a few pyrethroids, such as bifenthrin and lambda cyhalothrin [21].

In 2017, Minnesota and South and North Dakota were added to the list of states where pyrethroids failed to control soybean aphids in the field [78]. In field areas, where the soybean aphid population was not properly controlled by pyrethroids, it has been observed that living (i.e., presumably resistant) aphids have occasionally been seen in patches. This might be proof of a combination of aphid strains that have fluctuating insecticide susceptibility levels, thus settling the fields. The 2017 laboratory bioassays demonstrated that pyrethroid resistance in soybean aphids is a genuine phenomenon in the states of Minnesota and Iowa as well as in Manitoba, Canada [79].

In laboratory experiments conducted in China, soybean aphids were exposed for 40 generations to lambda-cyhalothrin. The results of the process demonstrated a 76-fold resistance to the insecticide used, in addition to the phenomenon of cross-resistance towards other organophosphates (such as chlorpyrifos and acephate), pyrethroids (such as cypermethrin, esfenvalerate, cyfluthrin, and bifenthrin), and the carbamate carbofuran [45]. The results are shown in Table 1. Like other insects, aphids use a wide variety of mechanisms (such as esterases and glutathione S-transferases; metabolic resistance mediated by monooxygenases; insensitivity of the target site, i.e., knockdown resistance (*kdr*) as well as super-*kdr* combined with a reduced cuticular penetration) that would be beneficial in overcoming pyrethroid insecticides [10].

Additional research in China has tabulated results showing that soybean aphids, by means of elevated esterase combined with cytochrome P450 expression, can overcome insecticides. This explains the phenomenon of cross-resistance witnessed by Xi et al. [45]. In the Upper Midwest of the United States, the prevalence of pyrethroid resistance has been observed in soybean aphid colonies [21]. A rapid management mechanism aimed at adequate soybean pest management can result in profitable soybean production [78]. 

The high mobility factor of winged soybean aphids is the reason why there is a considerable chance that insecticide-resistant soybean aphid populations may spread to fields in other soybean-producing areas [80]. In relation to the problems with agricultural yield and insecticide-resistant soybean aphids, consultants, growers, and applicators are urged to carefully consider and choose their soybean aphid control strategies. We have compiled a list of factors that may have played a vital role in the evolution of insecticide resistance, along with guidelines for the control of several soybean aphid populations that might be potentially resistant.

In order to tackle the challenge of insecticide-resistant strains of soybean aphid, numerous control policies are recommended for preventing the spread of resistance as well as crop loss due to subsequent pest-induced resistance. In order to minimize the selection pressure aimed at resistance development against insecticides, fields should be sprayed only when necessary. Starting at the vegetative development stage and continuing over the R5 stage of growth, it is imperative that soybean fields are scouted for soybean aphids regularly (every 7–10 days) [81,82]. Utilize the economic threshold level (ETL, i.e., over 250 aphids per plant in 80% of plants, with rising aphid numbers) to determine when to apply insecticides [78,83]. Studies have shown that the application of insecticides for soybean aphids beneath the ETL rarely offers commercial advantage, and pests will be inappropriately exposed to pesticide, enabling increased selection pressure [84]. Once the threshold has been attained, to secure the yield, keep treating the field for 5–7 days [83]. Subsequently, it is recommended to make use of “Speed Scouting” or similar processes as a supplementary productive method for scouting [82].

#### Pyrethroid Resistance Allied to Na-Channel (Para) Gene

DNA sequence analysis found unique sequence variations in pyrethroid-targeted Na-channel (para) genes. Heterozygous and homozygous aphid pyrethroid resistance were conferred by *kdr* and *skdr* variations in gene studies [64], and eight exon structural variations encoding an alternative amino acid were reportedly discovered in 15 aphid species: T929S and M957L in *Illinoia rubicola* (Oestland); T929S, V931A, and R980C in *Hayhurstia atriplicis* (L.); L925F, T929S, and M957L in *A. pisum*; I935M and I936V mutations in *M. persicae*; and L925F in *Euceraphis papyrifericola*, in addition to the *kdr* and *skdr* variations. Moreover, the samples from 10 species also revealed the mutations L925F, T929S, M957L, M957I, and R980C [65,66]. The M957L/I mutations were dominant in seven species, making mutant forms less likely and alternative forms more likely to be species-specific (Table 2). The detoxification enzymes glutathione S-transferases (GSTs) will be discussed in Section 3.4. in details. 

### 2.3. Carbamate Resistance

Certain insects’ resistance to carbamates has been linked to multiple resistance mechanisms. In the fall armyworm (*Spodoptera frugiperda*), microsomal cytochrome P-dependent monooxygenases were discovered to be the cause of carbaryl resistance [85]. Higher hydrolase activity was found to be a key reason why the German cockroach *Blatella germanica* and the *Myzus persicae* (Sulz.), (peach-potato aphid) became resistant to carbaryl [86,87]. 

In two of the *M. persicae* multilocus genotypes (MLGs) that were investigated, there was considerable pirimicarb resistance that was linked to the MACE mutation. Limited methamidophos resistance was also revealed, perhaps due to increased carboxylesterase function in certain MLGs. MLGs were likewise vulnerable to imidacloprid, whereas toxicity to pirimicarb and methamidophos was correlated. Studies have shown higher levels of resistance in various species of aphids, especially *M. persicae*, *A. gosypii*, *R. padi*, and *Nasonovia ribisnigri*. Insecticide resistance mutations to carbamates (*kdr* and MACE) were ranging from moderately high to extremely high levels, i.e., upto 4500-fold (Table 1) [41,42,43,44]. By using a larger MLG collection, from which a set of nine genotypes was chosen, were able to detect elevated carboxylesterase function in linkage disequilibrium in a manner unrelated to reproductive fitness or metabolic rate. Resistance to organophosphates was less common but dimethyl carbamates resistance were still present. This pirimicarb resistance may be due to the progressive replacement of organophosphate insecticides with pirimicarb and other chemical classes, as the resistance management approaches to aphids in a widespread area must be considered [88,89]. 

#### Mechanism of Carbamate Resistance

Carboxylesterases E4 and FE4 hydrolyze and sequester insecticide in the insect nervous system before reaching their target site [87,90]. Research discovered that overproduction is genetically rooted in E4 and FE4 gene amplification [59]. The gene copy number increasing by 4 times (maximum 80 copies) was demonstrated to be closely linked with the resistance phenotype, resulting in increasingly resistant phenotypes of aphids. E4 gene amplification was demonstrated by FISH using chromosomal translocation (the autosomal 1,3 translocation event) and a tandem array of head-to-tail amplicons located on autosome 3 at a single heterozygous position [91,92]. FE4 gene amplification, on the other hand, is not linked to a chromosomal rearrangement and can be found throughout the genome at multiple locations [91,93]. Revertant clones can lose esterase gene expression as well as pesticide resistance in the same generation, which is a fascinating component of resistance occurring after increased E4 genes [94]. The deletion of 5-methylcytosine (5 mC) within E4 genes in CpG doublets causes gene silence through demethylation in reverted individuals [95,96]. In the absence of pesticide selection, the production of esterase enzyme (esterase production is 3% of total protein) can be switched off in R3 aphids. Although the chromosomal rearrangement is obviously linked to E4 amplification, the specific mechanism by which this leads to E4 amplification is unknown. Even less is known about how FE4’s amplification occurs; speculation that inversions, reciprocal exchanges, or transposable elements may be involved remains unverified [97].

The acetylcholinesterase enzyme (AChE), a serine hydrolase that hydrolyzes the neurotransmitter acetylcholine at cholinergic synapses, is insensitive to inhibition, according to biochemical inhibition studies. When AChE (MACE) is altered, dimethyl carbamate pirimicarb is more than 100 times less sensitive to the affected insect [98]. Cloning of the AChe (ace) gene using homology of the *Drosophila melanogaster* and *Musca domestica* ace locus was the first effort to establish the molecular basis of MACE, but the study was unsuccessful in identifying the variations in amino acids of resistant and susceptible clones of aphids [60]. Studies indicated that a number of insects, along with *M. persicae*, contain two ace genes, and that in these species, ace-1, which encodes a pesticide target, differs from ace-2, which encodes an ortholog from *Drosophilla melanogaster* and *M. domestica* [62]. *M. persicae* ace-1 gene sequencing revealed that the insensitive enzyme’s predicted protein structure had one amino acid alteration, S431F, that was associated with pirimicarb resistance [61,63]. Following this, functional evidence showed that this mutation significantly alters the affinity of pirimicarb for AChE when expressed in recombinant form in aphids [99]. The position of ligands in the active site is determined by the S431F mutation of AChE’s acyl pocket. In order to inhibit pirimicarb from engaging with at least one catalytic triad member in the active site, it is projected that a modest serine would need to be replaced with a larger hydrophobic phenylalanine [61,63]. S431F substitution is significant since it is the exact opposite of what is seen in non-aphid insect species as well as in vertebrates, where greatly conserved phenylalanine is frequently found at this location in the AChE of *M. persicae* resistant to pirimicarb. There is strong evidence indicating that serine is the most common residue among wild-type aphid AChE, and mutation that causes pirimicarb insensitivity is the only type that alters the amino acid [63]. In addition, *kdr* (L1014F) mutation has been found to be associated with carbamate resistance (Table 2) [100].

Mutero et al. reported that substantial significant outcomes occur from four *D. melanogaster* mutations, elevating the fitness in the pesticide context (by lowering enzyme inhibition), and lowering the fitness without pesticide (via affecting enzyme stability and acetylcholine synthesis) [101]. A slight doubling of the AChE section of the genome inserts a vulnerable and resistant allele on one chromosome [102].

### 2.4. Neonicotinoid Resistance

Neonicotinoids account for around a quarter of the global pesticide market. Thiamethoxam and imidacloprid lead a powerful new class of chemical insecticides used to combat insect pests in a variety of agricultural crops [103]. Neonicotinoids’ nitro substituent influences their selectivity and potency for the nicotinic acetylcholine receptor in insects (nAChR). Seven commercialized neonicotinoids are linked to the production of more than 100 distinct metabolites in plants and mammals [104]. Contrary to expectations, neonicotinoids have remained highly effective against two important species of aphids, *A. gossypii* and *M. persicae*, after more than two decades of gradually increasing use. Neonicotinoid-resistant samples of *M. persicae* have been detected in Europe, the United States, and Japan (Table 1).

#### Mechanism of Neonicotinoid Resistance

Amplification of a particular P450 gene (CYP6CY3) was found to be at least partially responsible for a 22-fold increase in overexpression, as was demonstrated by quantitative PCR. Neonicotinoid resistance has been linked to reduced cuticular penetration, as demonstrated by the overexpression of multiple cuticular protein-encoding gene sequences (2–16 fold) in microarray analysis, as well as artificial feeding and penetration studies employing radiolabeled insecticide [67]. One mutation point was found in the loop D region nAChR β1 subunit (R81T) [68]. Additionally, in the resistant aphid clone, an arginine to threonine replacement was found in the loop D region of the nAChR 1 subunit, as evidenced by nAChR subunit (Mp1–5 and Mp1) genes (R81T) [105]. Carboxylesterase, MACE, and knockdown (*kdr*) pesticide resistance pathways are efficiently circumvented by neonicotinoids in this species (Table 2) [106]. R81T and V101I variants that are linked to resistance have been found in the imidacloprid-resistant *M. persicae* AH19 strain. This is the first description of the V101I variant [69]. The immidacoprid-resistant strain (IMI_R strain) of *A. gossypii* Glover has three target-site variations in the nicotinic acetylcholine receptor (nAChR) β1 subunit. According to reports, *A. gossypii* and *M. persicae* exhibit imidacloprid resistance due to the R81T mutant. V62I and the K264E mutations were found in *A. gossypii* first, and also found in filed strains, indicating their possible contribution to imidacloprid resistance in aphids [70].

## 3. Gene Expression Studies

The identification process of specific genes playing their role in insecticide detoxification and their genetic pathways is very valuable for the management and control of aphids. Moreover, using next-generation technologies for the purpose of revealing resistance and analyzing the genes related to insecticide using transcriptome profiles fills the gaps left by previous studies and provides us new concepts regarding insecticide resistance in aphids [107]. Yet, investigations of insecticide detoxification in aphids are limited. The most common method now being utilized to analyze gene expression is RNA-Seq [108], and a similar approach has also been employed to establish the genes associated with insecticide metabolism in numerous species, but there are very few transcriptome analyses regarding aphids in insecticide pressure that have been performed to date [109,110]. 

Several mechanisms that render individuals resilient to chemical insecticides, such as reduced cuticular penetration, mutations of targeted receptor sites, and increased levels of detoxification enzymes, are found in aphids [12,20]. The activation of genes is believed to be a fundamental mechanism conducting various phases of xenobiotic detoxification through which aphids counteract exposure to several insecticidal compounds [111]. Although the primary coping mechanism in aphids is detoxification, transcriptomic studies have made it clear that a higher degree of complexity is implicated in the response to insecticides, as it was supposed previously [111]. Under the selection pressure of insecticide toxicity, a major percentage of the aphid transcriptome is changed. Several hundreds of genes involved in a wide range of pathways are expressed differentially in multiple resistant genotypes [45,112].

### 3.1. Stress-Responsive Genes

As an example, the genes that respond to general stress that are allied with homeostasis renewal, such as peptidases and heat shock proteins, which interact with damaged proteins, are expressed differentially in aphids with insecticide stress [45,113,114]. The enhanced expression of cuticular proteins may function against insecticides as a first line of defense. The mechanism involves a reduction in permeability through the aphid exoskeleton. A comparative analysis of transcriptional polymorphism within genotypes with several resistance mechanisms highlighted that substantial plasticity in gene expression is exhibited by aphids after exposure to insecticides [114].

It is reported that analyzing up- or downregulation of specific enzyme sequencing in response to insecticide application or a receptor gene is the primary step in understanding enzymes involvement in pest resistance. According to unigenes analysis, the production of aphid regulation by a single gene does not lead to the tolerance of insecticides; instead, tolerance is a consequence of the combined regulation of several genes. The KEGG and GO pathways are increasingly prevalent as the number of DEUs increases with the duration of pesticide exposure. In fact, certain genes associated with insecticides, namely, glutathione S-transferase, carboxylesterase, acetylcholinesterase, acetylcholine receptor, chloride channel, and superoxide dismutase, were only differently expressed following a 36 h treatment with the insecticides [107].

### 3.2. Putative Insecticide Resistance-Related Genes

**Figure 1:**
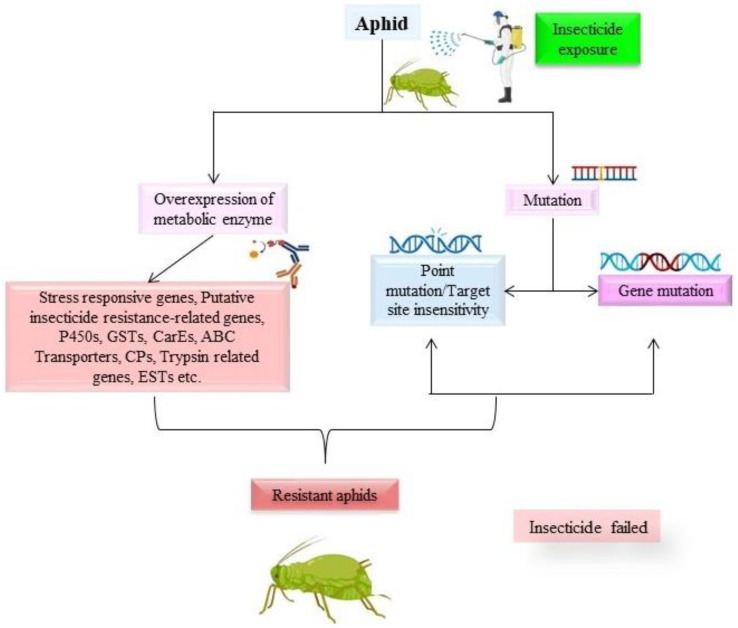
Illustration of the effects of insecticide exposure on aphids. The figure demonstrates how the insecticide fails and resistant aphids are produced.

According to the findings of a recent study, elevated esterase and cytochrome P450 (CYP) activity of an aphid population (reared and tested in laboratory) was selected for pyrethroid resistance [45]. This mechanism along with other mechanisms is described in Figure 1.

### 3.3. Cytochrome P450 (P450)

One of the enzymes that performs several metabolic roles is Cytochrome P450. Drug metabolism mediated by elevated levels of cytochrome P450 can be categorized as a major insect detoxification mechanism [115]. P450’s plays a significant role in a wide range of physiological processes including hormone metabolism, parasitic plant adaptation [116,117], and resistance to insecticides [118]. Furthermore, in *Culex pipiens* cytochrome, P450 is related to dichlorvos, propoxur, and pyrethroid insecticide resistance [119]. Overexpression of the P450 monooxygenase enzyme represents the most prevalent mechanism of chlorpyrifos and imidacloprid resistance. The enzyme P450 monooxygenase is essential for aphid resistance and insecticide detoxification [67,120,121].

The five CYP unigenes were also elevated to membership in the CYP 4 and 6 gene families. Interestingly, compared to other P450 families, these genes belong to the two families that are more commonly linked to insecticide resistance, thus playing key roles in the metabolism and in pesticide detoxification [115]. However, many single-copy genes in insects are part of the CYP2 clan as well as the mitochondrial CYP clan. Most multiple-copy paralogs are part of the CYP3 and CYP4 clans [122,123]. The majority of paralogs with more than one copy fall into the CYP3 and CYP4 clans [124]. Many tightly paralogous genes are members of P450 blooms lineage-specific family expansions [125]. It was supposed that the phenomenon of overexpression of unigenes in these two families was involved in *S. avenae* insecticide tolerance and detoxification. The nicotine- and neonicotinoid-resistant peach potato aphid (*M. persicae nicotianae*) has expanded a dinucleotide microsatellite in its CYP6CY3 promoter [126]. Aphid bacteriocytes (highly specialized aphid cells that contain *Buchnera aphidicola*, an obligatory endosymbiont that feeds its host necessary amino acids and other nutrition) are the primary locations of expression for CYP6CY3, according to recent research on this P450 in *M. persicae* [127]. The strain of *M. persicae nicotianae* that has acclimated to tobacco showed an increase in the expression of CYP6CY3 inside this tissue. Moreover, very high amounts of CYP6CY3 (>2500-fold in comparison to that in the guts of *M. persicae sensu stricto*) were found in this strain. These gene alterations seem to guard against the harmful or inhibiting effects of nicotine on that aphid host as well as its necessary symbiont [127]. Following exposure to imidacloprid, the CYP6FV12 gene in *B. odoriphaga* underwent various fold modifications at various stages of life. The relationship between the CYP6FV12 gene and *B. odoriphaga* resistance to imidacloprid has now been established [128]. In *Bemisia tabaci*, it was confirmed that CYP6CM1vQ was connected with a high level of imidacloprid resistance [129].

### 3.4. Glutathione S-Transferase (GST)

The detoxification enzymes referred to as glutathione S-transferases (GSTs) can conjugate electrophilic pesticide metabolites to glutathione for faster excretion [130,131], and GSTs, particularly those with a-methyl modifications, may boost OP resistance; they are also involved in pyrethroid resistance. Indeed, GSTs (EC 2.5.1.18) are a family of enzymes that have more than one function. They are involved mostly in the phase II metabolism of xenobiotic substrates and are found in many aerobic organisms [132,133]. They facilitate the coupling of electrophilic xenobiotics with reduced glutathione (GSH), which increases their water solubility and facilitates excretion [134,135,136,137]. Insect GSTs are the primary detoxifying enzyme and are of special importance due to their function in pesticide resistance [138,139]. GST enzymes are present in many insects and, being soluble and stable, their expression is stimulated by xenobiotic exposure [131], demonstrating that analogous gene regulatory mechanisms induced and overexpressed GSTs and P450s [51].

GSTs are liable to detoxification from xenobiotic compounds such as plant secondary metabolites and insecticides [140,141]. In insecticide resistance in insects, elevated levels of GST expression are crucial [105]. Moreover, cytosolic matrix GSTs in insects are categorized into at least six classes:

1. Delta
2. Epsilon
3. Omega
4. Sigma
5. Theta
6. Zeta

This is reliant on N-terminus sequence homology, immunoreactivity, substrate specificity, as well as sensitivity to diverse inhibitors [142]. The classes Epsilon and delta are distinct to insects. Delta, omega, sigma, theta, and zeta GST categories were identified in *Cinara pinitabulaeformis* Zhang et Zhang transcriptome. However, we were unable to recognize relevant genes from the epsilon category although mainly wingless aphids, such as pea aphid *A. pisum*, exhibit the GST delta class [143].

### 3.5. Carboxylesterase CarEs

Omethoate is a synthesized OP, and omethoate resistance was associated with higher CarEs expression in *Aphis gossypii* [144]. Carboxylesterase is one of the three enzymes whose overexpression causes metabolic resistance to insecticides. Omethoate is a synthetic OP; increased CarEs expression in *Aphis gossypii* was associated with omethoate tolerance [145].

### 3.6. ABC Transporters

Through phase III of the detoxification procedure, ABC transporters play an important role, transporting polar molecules or conjugates outside the cell. Chlorpyrifos resistance has been linked to ABC transporters. Eight subfamily transporter genes (ABCB/C/D/G) in *Laodelphax striatellus*-resistant strains of chlorpyrifos and imidacloprid, when compared with a susceptible strain, were significantly upregulated [146]. These results indicate that ABC transporters may be involved in *L. striatellus* resistance to numerous insecticides. Following a 48 h permethrin treatment, two of five ABC transporter genes examined in *Anopheles gambiae* showed downregulation [147]. Verapamil, an ABC inhibitor, greatly increased the toxicity of ABM, indicating that ABC may play a role in detoxification. The 30 ABC genes, which were all upregulated, may contribute to *C. pomonella* ABM resistance [148]. ABC transporters could be crucial in *S. avenae*’s ability to detoxify and its resistance to pesticides.

### 3.7. Cuticle Protein Genes

In *Culex pipiens* pallens, cuticle protein has an essential role for the development of deltamethrin resistance. CPLCG5 encoded a cuticle protein that contributed to pyrethroid resistance by causing stiffness and boosting cuticular density. Five cuticle protein genes were differentially expressed in deltamethrin-resistant *C. pipiens* pallens as compared to susceptible strains, and overexpression of precursors was identified, coupled with cuticle protein CP14.6, in a deltamethrin-resistant strain. This supports the idea that mosquitoes can fend off pesticides by cuticle regulation, ultimately resulting in cuticular resistance [149].

### 3.8. Trypsin-Related Genes

Under insecticide stress, many DEUs in *B. oriphaga* may be attributed to genes associated to Trypsin. Deltamethrin-resistant strains in *C. pipiens* pallens were found to be highly expressed. Trypsin expression in *Sodatella furcifera* was increased in response to exposure to triazophos, chlorpyrifos, imidacloprid, and abamectin [150].

### 3.9. Other Insecticide Resistance-Related Genes and Insecticide Receptors

In total, 187 unigenes have exhibited possible roles in pesticide resistance, detoxification, and pesticide receptors, including superoxide dismutase, carboxylesterase, acetyl-CoA carboxylase, sodium channel, ryanodine receptor, chloride channel, c-aminobutyric acid (GABA) receptors, nicotinic acetylcholine receptor, and acetylcholinesterase. Fundamental information for further understanding pesticide mechanisms can be provided by the study of these genes, which are not described in aphids [151].

## 4. Conclusions

This review of recent studies evaluated that several species of aphids have recently developed resistance against different classes of insesticides. This is one of the most important ecological changes that must be evaluated and resolved in a timely manner. To avert the proliferation of resistance in aphids, it is important to rotate between insecticides [152], with distinct mechanisms of action while focusing on the development of new eco-friendly insecticides. Therefore, genetic changes and the molecular basis of action of several insecticides was observed, leading to the conclusion that resistance mechanisms must be evaluated on a priority basis by entomologists. The ultimate goal of this study was to bring attention to the specific factors that play a role in this resistance and in the eradication of such problems, in order to encourage the implementation of eco-friendly and sustainable integrated pest management programs for aphids. In addition, this study aimed to impart this knowledge at the grassroots level while keeping in mind the impact of insecticides on non-target organisms. Thus, the determination of different types of resistance must be conducted with urgency in order to ease insecticide resistance management measures, such as genetic control combined with the release of susceptible strains.

## Figures and Tables

**Table 1:** Level of resistance in different species of aphids to various insecticide groups.

Species Name	Insecticide	Insecticide Group	Level of Resistance	References
*Aphis gossypii*, *Myzus persicae*, *A. fabae* and *Rhopalosiphum padi*	Methmidophos, monocrotophos, and phosphamidon	Organophosphates	1- to 85-fold	[39,40,41]
*M. persicae*, *A. gosypii*, *R. padi* and *Nasonovia ribisnigri*	Pirmicarb and Propoxur	Carbamates	up to 4500-fold	[41,42,43,44]
*M. persicae* and *A. glycines*	Lambda-cyhalothrin, bifenthrin and alpha-cypermethrin	Pyrethroids	up to 1000-fold	[21,45,46]
*M. persicae* and *A. gosypii*	Imidacloprid, nitenpyram, thiacloprid, acetamiprid, Clothianidin, Thiamethoxam, and Dinotefuran	Neonicotinoids	3- to 394-fold	[46,47,48,49,50]

**Table 2:** Mutation and genes involved in the insecticide resistance mechanism in aphids.

Insecticide	Mutation Involved	Enzyme/Gene Involved	References
Organophosphate	A128V, H104R, T333P, A302S, F139L, F368(290)L, S329(228)P, V435(356)A, Thr^210^→Met^210^, Asn^294^→Lys^294^, Gly^408^→Asp^408^ and Ser^441^→Phe^441^	Carboxylesterases, AChE genes (*Ace*1 and *Ace*2)	[41,56,57]
Carbamate	S431F, *kdr* (L1014F) mutation	AChE (*Ace*1 and *Ace*2), GST, carboxylesterases, E4, or FE4	[59,60,61,62,63]
Pyrethroids	*kdr* (L1014 and M918 mutation), I935M, I936V, L925F, M957I, M957L, R980C, T929S and V931A	Na-gated channel, P450s	[64,65,66]
Neonicotinoids	R81T, arginine to threonine mutation, V101I and V62I, K264E	P450 gene (CYP6CY3), Carboxylesterase, MACE and nicotinic acetylcholine receptor (nAChR) β1 subunit	[12,67,68,69,70]

## Data Availability

The study did not report any data.
